# Diverse Localization and Protein Binding Abilities of Glyceraldehyde-3-Phosphate Dehydrogenase in Pathogenic Bacteria: The Key to its Multifunctionality?

**DOI:** 10.3389/fcimb.2020.00089

**Published:** 2020-03-03

**Authors:** Monika Kopeckova, Ivona Pavkova, Jiri Stulik

**Affiliations:** Department of Molecular Pathology and Biology, Faculty of Military Health Science, University of Defence, Hradec Kralove, Czechia

**Keywords:** glyceraldehyde-3-phosphate dehydrogenase, pathogenic bacteria, protein-protein interaction (PPI), moonlighting proteins, localization

## Abstract

Bacterial proteins exhibiting two or more unrelated functions, referred to as moonlighting proteins, are suggested to contribute to full virulence manifestation in pathogens. An expanding number of published studies have revealed the glycolytic enzyme glyceraldehyde-3-phosphate dehydrogenase (GAPDH) to be a multitasking protein with virulence impact in a number of pathogenic bacteria. This protein can be detected on the bacterial surface or outside the bacterial cell, where it interacts with host proteins. In this way, GAPDH is able to modulate various pathogenic processes. Moreover, it has been shown to be involved in non-enzymatic processes inside the bacterial cell. In this mini review, we summarize main findings concerning the multiple localization and protein interactions of GAPDH derived from bacterial pathogens of humans. We also briefly discuss problems associated with using GAPDH as a vaccine antigen and endeavor to inspire further research to fill gaps in the existing knowledge.

## Glyceraldehyde-3-phosphate dehydrogenase in bacterial pathogenesis

Although bacterial glyceraldehyde-3-phosphate dehydrogenase (GAPDH) is a classic glycolytic enzyme catalyzing the conversion of glyceraldehyde-3-phosphate to 1, 3-bisphosphoglycerate (Stone et al., 1985), independent studies from various laboratories have demonstrated additional roles of GAPDH unrelated to its enzymatic function. Accordingly, in addition to its cytosolic localization, GAPDH has been detected on the bacterial cell surface or as a secreted protein in many bacteria. The GAPDH sequence nevertheless lacks any known recognition motif for extracytosolic trafficking (e.g., N-terminal signal sequence, hexapeptide sorting motif, or C-terminal hydrophobic tail) (Pancholi and Chhatwal, 2003) and the mechanism of its translocation remains open to discussion. A number of studies have revealed that bacterial GAPDH is able to interact with intracellular bacterial proteins and/or several host proteins outside the bacterium, thus indicating its multifunctional character (Henderson and Martin, 2011; Sirover, 2011; Boradia et al., 2014a,b). Such proteins whose two or more independent functions are associated with a single polypeptide chain are referred to as moonlighting proteins (Pancholi and Fischetti, 1992; Jeffery, 2009). Moreover, the non-enzymatic functions of GAPDH have been shown to play important roles within pathogenic processes in many bacteria (Henderson and Martin, 2011). Our understanding of the biological function and role of GAPDH in pathogenesis is nevertheless far from complete.

## Multiple Localization of GAPDH

The cell surface localization and/or secretion of GAPDH involved in its non-enzymatic activities also have been demonstrated in bacterial pathogens of humans, including *Escherichia coli, Francisella tularensis, Mycobacterium tuberculosi*s, *Mycoplasma genitalium, Mycoplasma pneumoniae, Staphylococcus aureus, Streptococcus agalactiae, Streptococcus pyogenes*, and *Streptococcus pneumoniae*. This allows GAPDH to interact with proteins of the host organism. Because GAPDH is capable of binding, for example, to the human serum proteins, extracellular matrix proteins, cytoskeleton proteins, and others (Table 1), it can be expected to contribute to virulence (Alvarez et al., 2003; Egea et al., 2007; Alvarez-Dominguez et al., 2008; Fugier et al., 2009; Matta et al., 2010; Dumke et al., 2011; Terrasse et al., 2012; Gao et al., 2014; Pavkova et al., 2017; Querol-García et al., 2017). Interestingly this unusual localization seems not to interfere with its enzymatic activity (Pancholi and Fischetti, 1992; Egea et al., 2007). Using immunoelectron microscopy, some studies have provided visual proof of the cell wall location of GAPDH (Egea et al., 2007; Pavkova et al., 2017). But how can a cytoplasmic protein without a signal sequence or other sorting motif leave the intracellular space and eventually anchor onto the cell surface? A general mechanism for this process remains obscure.

**Table 1:** Binding of bacterial GAPDH to host proteins.

**Bacteria**	**Host target**	**Extra/Intracellular**	**Reference(s)**
*Escherichia coli*	Fibrinogen Plasminogen	Extra	Egea et al., 2007
*Francisella tularensis*	Fibrinogen Fibronectin Plasminogen	Intra	Pavkova et al., 2017
*Listeria monocytogenes*	Rab5a	Intra	Alvarez-Dominguez et al., 2008
*Mycobacterium tuberculosis*	Lactoferrin Transferrin	Intra	Boradia et al., 2014a,b; Malhotra et al., 2017
*Mycoplasma genitalium*	Mucin	Extra	Alvarez et al., 2003
*Mycoplasma pneumoniae*	Fibrinogen Lactoferrin Laminin Vitronectin Fibronectin	Extra	Dumke et al., 2011; Gründel et al., 2016; Grimmer and Dumke, 2019
*Staphylococcus aureus*	Plasminogen Fibrinogen Vitronectin Transferrin	Extra	Modun and Williams, 1999; Taylor and Heinrichs, 2002; Ebner et al., 2016
*Streptococcus agalactiae*	Fibrinogen Laminin Mucin Plasminogen Transferrin	Extra	Magalhães et al., 2007; Nagarajan et al., 2019
*Streptococcus pyogenes*	C5a anaphylatoxin Actin Fibronectin Lysozyme Myosin Plasminogen/Plasmin	Extra	Pancholi and Fischetti, 1992; Winram and Lottenberg, 1996; D'Costa and Boyle, 2000; Terao et al., 2006
*Streptococcus pneumoniae*	C1q protein Hemoglobin Heme Plasminogen/Plasmin	Extra	Bergmann et al., 2004; Terrasse et al., 2012; Vázquez-Zamorano et al., 2014

Even though several studies have endeavored to clarify this phenomenon, they have not come up with a clear answer. Inasmuch as the bacteria differ from one another in their membrane structures and secretion efficiency, they may use different pathways to export proteins without a signal sequence to the exterior of the cell (Wang et al., 2014; Green and Mecsas, 2016). So, while some researchers suppose the release of GAPDH is due to cell lysis, others have suggested the involvement of specific secretion processes. Pasztor et al. (2010) have reported that several cytoplasmic proteins of *Staphylococcal aureus*, including GAPDH, move into the culture supernatant due to bacterial lysis induced by the major autolysin Atl. Secretome analysis of relevant mutant strains has shown that the secretion of GAPDH was scarcely detectable in the *atl* mutant strain, whereas in the *tagO* mutant strain, characterized by an increased cell lysis profile, the amount of GAPDH in the supernatant was significantly increased compared to the amount seen in the wild-type strain. Similar observation has also been reported for some *Streptococcus* spp. In *Streptococcus pneumoniae*, larger quantities of GAPDH were detectable on the bacterial surface of the wild-type strain compared to *lytA* mutant strain, which does not lyse during the stationary phase (Terrasse et al., 2012). Similarly, penicillin G-induced cell lysis of *Group B Streptococcus* significantly increased the surface display and secretion of GAPDH into the culture supernatant; in contrast mutant strains with decreased rates of lysis revealed reduced amounts of surface GAPDH (Oliveira et al., 2012).

On the contrary, Boël et al. (2005) provided strong evidence that surface-displayed GAPDH of *S. pyogenes* is derived from a still unknown secretion system. They fused a hydrophobic tail to the C-terminal end, which resulted in GAPDH retention within the cytosol. Thereby, they confirmed the importance of the GAPDH C-terminus for its export out of the bacteria (Boël et al., 2005). Moreover, the redirection of GAPDH from surface to cytosol led to impaired bacterial adherence and antiphagocytic activities and was also accompanied by global changes in gene expression, including the downregulation of important virulence factors like exotoxins or streptolysin (Hong et al., 2011). All these findings indicate that extracellularly localized GAPDH plays an important role in virulence of *S. pyogenes*. Depending on growth conditions of enteropathogenic *Escherichia coli* (EPEC), two distinct pathways participate in GAPDH secretion. In cells grown in Dulbecco's modified Eagle's medium, the GAPDH secretion was mediated by the type III secretion system generally involved in translocation of various effector proteins into the infected cells. Additionally, the interaction of GAPDH with CesT, a specific chaperone for type III effectors, was established. The associated chaperone may stabilize the GAPDH molecules and prevent them from interacting with other cytosolic partners, thus enabling their targeting to the type III secretion apparatus. The other secretory pathway has not been further described and is responsible for GAPDH secretion in EPEC and in probiotic *E. coli* strains grown in Lysogeny broth medium (Aguilera et al., 2012). The nonpathogenic strains do not encode the type III secretion system components, and thus they are not able to secrete GAPDH when grown in Dulbecco's modified Eagle's medium. These strains may profit from the interaction of GAPDH with extracellular host proteins during gut colonization.

Another hypothesis supposes that GAPDH trafficking may be associated with its posttranslational modification. This has been widely explored in eukaryotic cells (Ganapathy-Kanniappan, 2017). Only a few studies have been directed to posttranslational modifications of bacterial GAPDH, and their role in the extracellular export have so far not been investigated (Pancholi and Fischetti, 1992; Sun et al., 2010; Aguilera et al., 2012). The export mechanisms involved in GAPDH surface display or secretion outside the bacterial cells remain altogether unexplained for most of the pathogenic bacteria and will be subjects of future research.

## Protein–Protein Interactions of Bacterial GAPDH

Because protein-protein interactions play a key role in the function of predicting targeted proteins (Rao et al., 2014), knowledge of these may be valuable for elucidating potential non-enzymatic functions of bacterial GAPDH. Most of the published studies in this area have established GAPDH's binding capacity to certain host proteins impacting on a pathogen's adhesion and invasion (Table 1) and only a few of these have been focused on interactions of GAPDH with proteins within bacteria.

### Interactions With Intracellular Bacterial Proteins

Beyond confirming GAPDH's multifunctionality, the identification of interaction partners outside the glycolytic pathway might also contribute to deeper insights into biological processes inside the bacterial cell. Both Boël et al. (2005) and Hong et al. (2011) have indicated that bacterial GAPDH might contribute to the regulation of gene expression. From large-scale protein–protein interaction analyses performed in *E. coli*, it is evident that GAPDH interacts with a range of proteins, including metabolic enzymes and proteins involved in transcription or protein synthesis (Butland et al., 2005; Arifuzzaman et al., 2006). The only study to date aimed directly at identifying intracellular interacting partners of bacterial GAPDH was published by Ferreira et al. (2013). Immunoaffinity purification followed by mass spectrometry enabled the detection of several proteins involved in various cellular pathways, including metabolism, protein synthesis and folding, and DNA repair. For further verification and functional analyses, Ferreira et al. (2013) selected phosphoglycolate phosphatase (Gph), an enzyme involved in DNA repair of 3′-phosphoglycolate ends caused by oxidative stress mediated by the radiomimetic agent bleomycin (Povirk, 1996; Pellicer et al., 2003). Further observations have pointed to a role of bacterial DNA repair machinery also in *E. coli* (Ferreira et al., 2015). GAPDH was shown to interact with other repair enzymes AP-endonuclease Endo IV and uracil DNA glycosylase. Moreover, GAPDH deficiency was shown to result in increased sensitivity to the DNA damaging agents bleomycin and methanesulfonate. It is thus obvious that GAPDH might participate in DNA repair processes, but, once again, its actual functional role remains to be clarified.

### Interaction With Host Targets

Much more attention has been paid to the interactions of bacterial GAPDH with host targets. The surface-localized GAPDH of many pathogenic bacteria binds to various components related to extracellular matrix (ECM). Its binding specificity differs among individual pathogens, but plasminogen is the most common target (Table 1). Pathogens invade the host fibrinolytic system by recruiting host plasminogen on the cell surface and converting it to proteolytic plasmin, which plays a pivotal role in degradation of ECM proteins and fibrin clots (fibrinolysis) (Peetermans et al., 2016). The disruption of tissue barriers formed by ECM, basement membrane, and fibrin clots enhances bacterial dissemination through the host organism (Bhattacharya et al., 2012). It is thus one of the most important factors in the pathogenesis of bacterial infection. Targeting of the host cytoskeletal proteins actin or myosin by *S. pyogenes* GAPDH might allow the pathogen to invade and persist inside the host cells (Seidler and Seidler, 2013). There are many studies that document the binding of bacterial GAPHD to ECM host components. Although most of these studies merely identify one or more of several host binding proteins, only a few studies characterized the binding mechanisms or key residues in the GAPDH structure involved in the interaction (Gründel et al., 2016; Grimmer and Dumke, 2019; Nagarajan et al., 2019). As the spectrum of tested proteins varies between the studies and the binding affinities are not provided in most cases it is impossible to compare the GAPDH binding characteristics across all bacteria. The real functional impacts of these interactions are only assumed from the knowledge and require further research.

The ability of an invading bacterial pathogen to survive and proliferate within a host organism also depends on the availability of several trace elements, such as iron, an essential cofactor for diverse biochemical reactions. In a healthy mammalian organism, almost all the iron is bound to the transport proteins transferrin or lactoferrin or is stored in ferritin, because free iron catalyzes the production of toxic free oxygen radicals. The free ionic iron both in extracellular fluids and inside the cells is thus far too low to support bacterial growth. Bacterial pathogens have developed several strategies, however, to exploit iron from those iron-binding proteins (Cornelissen and Sparling, 1994; Modun and Williams, 1999; Rodriguez and Smith, 2003; Tullius et al., 2011). As do many other pathogenic bacteria, *Mycobacterium tuberculosis* secrets iron-binding molecules known as siderophores (e.g., mycobactin and carboxymycobactin) that compete with the host's iron-transport proteins (De Voss et al., 2000; Ryndak et al., 2010; Banerjee et al., 2011). Recently, a previously unknown alternative pathway independent of the siderophores was presented by Boradia et al. (2014a) wherein *M. tuberculosis* utilizes surface-localized GAPDH to capture the human transferrin and then internalizes the transferrin–GAPDH complex. Three years later, the same research group (Malhotra et al., 2017) reported that surface-localized GAPDH of *M. tuberculosis* has even greater binding affinity to lactoferrin. *M. tuberculosis* GAPDH thus acts as dual receptor for both transferrin and lactoferrin. The ability of surface-localized GAPDH to bind transferrin has newly been demonstrated for *Streptococcus agalactiae* (Nagarajan et al., 2019). Controversial data were published regarding the transferrin-binding cell wall GAPDH in *Staphylococcus* ssp. Whereas, Modun and Williams (1999) identified GAPDH as a staphylococcal transferrin-binding protein, further analysis performed by another group could detect no affinity of staphylococcal GAPDH for human transferrin. For this pathogen, the role of GAPDH in iron acquisition by transferrin should thus be interpreted with caution and more-detailed experimental evidence is needed in this regard (Modun and Williams, 1999; Taylor and Heinrichs, 2002). *Streptococcus pneumoniae* utilizes hemoglobin or heme instead of transferrin or lactoferrin as an iron source. GAPDH of this pathogen can bind both these proteins and has been proposed to participate in iron scavenging for bacterial needs (Yang et al., 2016).

Extracellular localization of GAPDH has also been demonstrated for bacteria with intracellular life cycles. One can therefore assume that intracellular bacteria might use GAPDH for manipulating some host cellular processes in order to customize the host cell milieu for their successful survival and proliferation. So far, the only study supporting this hypothesis was performed on intracellular Gram-positive *Listeria monocytogenes*. The surface-localized GAPDH interferes with the host Rab5a protein (Alvarez-Dominguez et al., 2008). The Rab proteins are small, monomeric GTPases/GTP-binding proteins from the Ras superfamily implicated in various cellular functions, including growth, protein trafficking, transmembrane signal transduction, and targeting and fusion of membrane bound organelles (Bhuin and Roy, 2014). *Listeria monocytogenes* escapes rapidly from the phagosomal compartment to the cytosol, where it replicates (Vázquez-Boland et al., 2001). Within the phagosome, the *L. monocytogenes* GAPDH evidently has the ability to ADP-ribosylate the Rab5a protein, thus blocking its function in phagosome–endosome fusion. As a consequence of this strategy, *L. monocytogenes* delays the phagosome maturation and gains time for escape from the vacuole prior to its fusion with endolysosome that would result in the pathogen's destruction (Alvarez-Dominguez et al., 2008).

GAPDH derived from extracellular pathogens seems to affect host cellular processes, too, as GAPDH secreted by *S. agalactiae, S. pyogenes*, and *Staphylococcus aureus* was reported to induce apoptosis in murine macrophages (Oliveira et al., 2012). To date, however, there are no further analyses elucidating the mechanisms responsible for this phenomenon.

Immunomodulatory activities of surface proteins represent another strategy for promoting a pathogen's survival in its host organism. *Streptococcus pyogenes* is one of few known bacteria able to bind and inhibit the C5a component of the complement system. As an integral part of the innate immunity, the complement system acts in early defense against pathogens prior to the activation of acquired immune response. It promotes cell killing by the formation of a membrane attack complex and production of molecules that stimulate the function of phagocytic cells and contribute to the inflammation manifestation. The C5a component is a potent anaphylatoxin and chemoattractant for neutrophils and macrophages (Kajita and Hugli, 1990). GAPDH exposed on the surface of streptococci captures the C5a, which is then degraded by surface-localized protease ScpA, resulting in disturbed chemotaxis and H_2_O_2_ production in neutrophils (Terao et al., 2006). In *S. agalactiae*, the secreted GAPDH exerts a stimulatory effect on B lymphocytes and to a lesser extent also on T cells. GAPDH-induced production of the anti-inflammatory cytokine interleukin-10 suppresses neutrophil recruitment in mice, which finally contributes to successful host colonization by this pathogen (Madureira et al., 2007, 2011).

## GAPDH as a Vaccine Candidate

Surface localization plus its role in virulence together with the binding to ECM proteins make GAPDH a suitable vaccine candidate for preventing infectious diseases. The host immune responses triggered by bacterial GAPDH have been studied extensively, and as a vaccine component GAPDH was able to induce protection against several pathogenic bacteria (Argiro et al., 2000; Ling et al., 2004; Liu et al., 2005; Perez-Casal and Potter, 2016). Focusing on human pathogens only, immunoprotective efficacy of GAPDH has been demonstrated in animal models against *S. agalactiae* (Madureira et al., 2011), *S. pneumoniae* (Sun et al., 2017), *Bacillus anthracis* (Matta et al., 2010), and *L. monocytogenes* (Calderon-Gonzalez et al., 2014). Regarding *L. monocytogenes*, the GAPDH peptide was tested as a component of a cell-based vaccine against listeriosis. Dendritic cells were used as adjuvants for immunostimulation. Despite those promising results, the high GAPDH sequence homology between the bacterial strains and humans should be taken into consideration in vaccine development. Homologous sequences can be responsible for inducing cross-immune responses that result in deleterious effects on human health (Perez-Casal and Potter, 2016; Franco-Serrano et al., 2018). A possible solution is the usage of immunoreactive epitopes present only in the pathogen's GAPDH for constructing an effective and safe vaccine instead of using the whole protein (Perez-Casal and Potter, 2016). Based on this strategy, Razim et al. (2018) decided to test selected epitopes for potential cross-reactivity. However, sequence alignment revealed high conservation of both peptides among selected pathogenic and commensal bacterial strains and some regions were even shared with the human equivalent. Consequently, the epitopes showed cross-reactivity with sera from patients infected with *S. agalactiae* or suffering from autoimmune diseases which excludes as potential candidates for a subunit vaccine (Razim et al., 2018). This experimental study demonstrated the real risks of a subunit vaccine based on moonlighting proteins and confirms to some extent the hypothesis published by Franco-Serrano et al. (2018), who questioned the suitability of such highly conserved proteins as vaccine antigens.

## Summary

**Figure 1:**
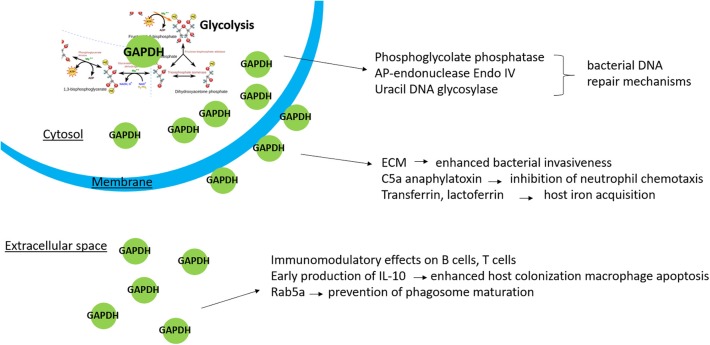
Non-enzymatic functions of bacterial GAPDH in relation to its localization.

In summary, it is evident that our current knowledge concerning bacterial GAPDH remains quite limited and provides much space for further research. It is indisputable that this protein has other functions unrelated to its enzymatic role, and it meets the criteria to be included into the family of moonlighting proteins. Data from previously published studies indicate that multiply localized GAPDH participates in other non-glycolytic processes (Figure 1): Cytosolic GAPDH is probably associated with DNA repair mechanisms, surface-exposed GAPDH seems to enhance bacterial invasiveness, and secreted GAPDH might directly affect the host's cellular pathways, including those related to the innate immune response. The non-enzymatic functions of GAPDH mostly have been inferred from its ability to bind certain proteins with known function. With few exceptions, the existing studies did not determine the precise functional roles of GAPDH in these interactions. In pathogenic bacteria it might be very difficult to distinguish between the pleiotropic effects due to its key position in glycolysis and the additional non-enzymatic functions. These facts should be taken into account prior to designating GAPDH as a virulence factor. The importance of GAPDH as a vaccine candidate remains questionable as well: On the one hand, the surface localization is associated with desirable immunoreactivity. On the other hand, the structural conservation significantly limits its application as a vaccine antigen. Despite this, investigation of GAPDH's multifunctionality is of great importance. Such additional studies might contribute to elucidating the mechanisms of bacterial pathogenesis essential for developing effective and safe therapeutic and prophylactic agents.

## Author Contributions

All authors listed have made a substantial, direct and intellectual contribution to the work, and approved it for publication.

### Conflict of Interest

The authors declare that the research was conducted in the absence of any commercial or financial relationships that could be construed as a potential conflict of interest.
